# In the eyes of the beholder: Race, place and health

**DOI:** 10.3389/fpubh.2022.920637

**Published:** 2022-08-12

**Authors:** Alfredo J. Velasquez, Jason A. Douglas, Fangqi Guo, Jennifer W. Robinette

**Affiliations:** ^1^Department of Psychology, Chapman University, Orange, CA, United States; ^2^Department of Health Sciences, Chapman University, Orange, CA, United States

**Keywords:** race/ethnicity, neighborhood physical disorder, self-rated health, perceived neighborhood safety, vulnerability

## Abstract

Racial and ethnic health disparities are fundamentally connected to neighborhood quality. For example, as a result of historical systemic inequities, racial and ethnic minorities are more likely to live in neighborhoods with signs of physical disorder (e.g., graffiti, vandalism), and physically disordered environments have been noted to associate with increased risk for chronic illness. Degree of exposure to neighborhood disorder may alter peoples' perception of their neighborhoods, however, with those most exposed (e.g., historically marginalized racial/ethnic groups) perhaps perceiving less threat from signs of neighborhood disorder. The purpose of the present study was to examine the complex interrelationships between people and place by investigating whether exposure to neighborhood physical disorder relates to residents' (1) perceptions of neighborhood safety and (2) perceptions of their health, and (3) examining whether these links vary by race/ethnicity. Using 2016–2018 Health and Retirement Study (HRS) data, a representative sample of US adults aged 51 years and older (*n* = 9,080, mean age 68 years), we conducted a series of weighted linear regressions to examine the role of neighborhood disorder in relation to both perceived neighborhood safety and self-rated health. Results indicated that greater neighborhood physical disorder was statistically significantly related to feeling less safe among non-Hispanic Whites and Hispanics, but not non-Hispanic Blacks. Regarding self-rated health, neighborhood physical disorder was statistically significantly related to poorer health among all racial/ethnic groups. These findings suggest that, despite differential interpretation of neighborhood disorder as a threat to safety, this modifiable aspect of peoples' environment is related to poor health regardless of one's race/ethnicity.

## Introduction

According to the World Health Organization (1), the conditions in which people grow up, live, and age are linked to health. Many forms of institutionalized discriminatory practices have contributed to neighborhood disparities where members of marginalized racial/ethnic groups live with more disadvantage in their neighborhoods (2–5). Furthermore, racial/ethnic disparities exist for many health outcomes, whereby non-Hispanic Whites often have better health than those of other racial/ethnic groups (6–8). Moreover, some evidence suggests that racial/ethnic health disparities are no longer evident once socioeconomic factors are considered (9). Critically, neighborhood disadvantage (e.g., poverty, disorder) is associated with poor health among residents (10–12). Researchers have long theorized that economic disinvestment in specific residential areas often leads to the deterioration of those areas, including an increase in signs of neighborhood physical disorder. Neighborhood physical disorder is, in turn, considered the more proximal factor that relates more directly to health than socioeconomic status (SES) (11). Although not the focus of the present study, social aspects of the neighborhood, such as crime, drug use, and loitering, have documented relationships with residents' health as well (3, 4) As such, the neighborhood features with known associations with health, such as neighborhood physical disorder (i.e., neighborhoods with more vandalism, litter, and run-down buildings and property) (4, 11, 13, 14), may be the starting point for understanding persistent connections between racial and ethnic health disparities and place (15–17).

Neighborhoods exhibiting signs of physical disorder, evidenced by signs of decay and lack of social controls (e.g., litter, vandalism), are related to people's withdrawal from public spaces and subsequent health problems (3, 12, 18). Some evidence suggests that neighborhood physical disorder is more normative of marginalized racial/ethnic groups compared to non-Hispanic Whites (19). In fact, some researchers have observed racial/ethnic differences in the report of, and concern about signs of neighborhood disorder (20, 21). For example, when asking a diverse sample of participants about signs of physical disorder in their neighborhoods, researchers observed that non-Hispanic Whites were more likely than other racial/ethnic groups, particularly non-Hispanic Blacks, to report such problems in their neighborhoods (22). This observation suggests that greater exposure to neighborhood disorder may result in different schematic processes whereby neighborhood disorder is more psychological distressing for those least exposed to it. One seemingly obvious question, then, is whether there are racial/ethnic differences in the relationship between neighborhood physical disorder and health.

Few existing studies investigate racial/ethnic differences in links between neighborhood physical disorder and residents' racial and ethnic characteristics, and none to our knowledge investigate health outcomes. Further, the paucity of research examining perceived disorder by racial and ethnic characteristics has been inconclusive. In one investigation, perceived neighborhood disorder was related to greater depletion of personal control, or the perception that one has control over their life outcomes (23). This relationship was greater among non-Hispanic Whites than non-Whites, however. The authors attributed this racial difference to a greater mismatch between personal and neighborhood statuses (e.g., SES) among non-Hispanic Whites compared to non-Hispanic Blacks, with this mismatch leading to greater cognitive dissonance among non-Hispanic Whites. The authors coined the term “Status Discord” to describe this phenomenon (23). Others have similarly posited that neighborhood disorder may be more detrimental to non-Hispanic Whites than non-Hispanic Blacks, but with a different rationale (24). The “epidemiological paradox” is described as a process by which marginalized racial/ethnic groups, given their greater exposure to disordered neighborhoods, develop greater coping and resilience and may thus fare better than their non-Hispanic White counterparts (24). In a more recent investigation, greater perceived neighborhood disorder was related to a depletion of personal mastery, or the belief that life chances are under a degree of personal influence (25). Although this investigation also observed a racial difference, the direction was markedly different; the disorder-mastery link was more evident among non-Hispanic Blacks than non-Hispanic Whites. Gilster (25) argued that the racial/ethnic difference observed in the disorder-mastery link can be explained by a situation of compound risk, or that non-Whites experience a greater accumulation of personal and neighborhood stressors than do non-Hispanic Whites. The Stress Process Model has long posited that those with fewer personal resources, those with more chronic or repeated stressors, and certainly those with both, will fare the worse health outcomes than those with more resources or fewer stressors. The Stress Process Model may thus be a suitable model to inform our understanding of when and how neighborhood physical disorder may be worse for non-Whites than non-Hispanic Whites (26).

Yet, some studies have reported no racial/ethnic differences in links between neighborhood features and indices of health (smoking, drinking, depression & walking behaviors) (27). The paucity of research on potential racial/ethnic differences in relationships between neighborhood physical disorder and health creates a critical gap in our understanding of health disparities. For example, scholars such as Millar (24) have argued that “taken together, there are limited and inconsistent findings on the relationship between neighborhood-level contexts and health and health-promoting behaviors across race and ethnicity.” The goals of the present study are both theoretically and policy motivated. The theoretical goal of the present analyses is to further characterize the subjective experience of neighborhood disorder among a diverse group of older US adults. An additional project goal is to identify differential vulnerability to physical disorder, a modifiable aspect of peoples' neighborhoods, among various racial/ethnic groups to inform policies that attend to neighborhood-level contextual conditions, focusing here on trash, vandalism, and safety concerns. As such, using national data from the Health and Retirement Study (HRS), this study investigated both perceptions of neighborhood safety and self-rated health in the context of neighborhood physical disorder as rated by third-party surveyors. The hypothesized relationships were assessed separately by groups of people self-identifying as non-Hispanic White, non-Hispanic Black, and Hispanic. Specifically:

1. To what extent does neighborhood physical disorder relate to residents' perceptions of safety in their neighborhoods?
2. To what extent does neighborhood physical disorder relate to residents' perceptions of their health?
3. To what extent do the above hypothesized links differ by race/ethnicity?

## Materials and methods

### Data

The HRS is a nationally representative sample of more than 20,000 US men and women aged 51 years and older, with data collected biannually since 1992 (28). HRS recruited households using a four-stage area probability sample design (29). The purpose of the HRS study is to examine the socioeconomic and health status of the older US population. HRS introduced enhanced face-to-face interviews (EFTF) in 2006 with a random half of the sample, and completed the EFTF with the other half in 2008. The EFTF allows for HRS interviewers to survey the area surrounding the participants' homes, as well as to leave behind a psychosocial questionnaire asking for participants' perceptions of neighborhood safety. HRS health records are linked to contextual data resources (CDR) that involves multiple sources of data, such as the American Community Survey (ACS) used in the current analyses (30). The present study uses the most recent 2016/2018 waves of HRS data to investigate relationships between HRS interviewer ratings of neighborhood physical disorder on participants' perceptions of neighborhood safety and self-rated health. Participants signed consent forms prior to data collection and research procedures were approved by the University of Michigan's Institutional Review Board.

### Measures

#### Perceived neighborhood safety

HRS participants answered a single item from the psychosocial leave-behind questionnaire, “People feel safe walking alone in this area after dark” (31). Responses ranged from 1 to 7, and these values were reverse-coded such that higher values indicated feeling safer.

#### Self-rated health

Health and Retirement Study participants reported their health with the following item, “Would you say your health is excellent, very good, good, fair, or poor?” These responses ranged from 1 to 5 and were revere-coded so that higher values indicated better health.

#### Race/ethnicity

Racial/ethnic categories were coded as the following: non-Hispanic Whites (1), non-Hispanic Blacks (2), and Hispanics (3).

#### Neighborhood physical disorder

Health and Retirement Study interviewers indicated whether the following were present within sight of the participant homes: vandalism, trash/litter/junk in the street/road, trash/litter/junk around the buildings in neighborhood, abandoned cars, and rundown yards. These items were coded as 0 = not present or 1 = present. A neighborhood physical disorder scale was then constructed by summing across the five indicators, with higher scores indicating more neighborhood physical disorder.

#### Covariates

Three census tract-level variables were included as covariates and were available *via* the ACS 2012–2016 5-year estimates (30). First, concentrated disadvantage was constructed by averaging three standardized scores: proportion unemployed, proportion female-headed households, and proportion in poverty. This measure adjusts for area SES to demonstrate the disorder-health link above and beyond associations with area SES. In order to achieve this, each of the three individual scores were standardized from the mean. Next, population density was defined as the total population per square mile. Lastly, racial/ethnic diversity was constructed by subtracting from the total population the proportions of the following racial/ethnic groups: non-Hispanic White, non-Hispanic Black, non-Hispanic Asian, non-Hispanic “Other,” and Hispanic (32). Several individual-level sociodemographic variables from the 2016 HRS tracker file were included as covariates. Age was coded in years and sex was coded as 0 = men and 1 = women. Education was coded as the following: 0 = no degree, 1 = General Educational Development (GED), 2 = high school diploma, 3 = 2-year college degree, 4 = 4-year college degree, 5 = master's degree, 6 = professional degree (Ph.D., M.D., J.D).

### Statistical analysis

To account for HRS's complex survey design, weighted analyses were conducted in Stata 17 using the svy: suite of commands. A series of linear regressions stratified by race/ethnicity examined the hypothesized relationships between neighborhood physical disorder and the two outcomes, perceived neighborhood safety and self-rated health. The first model investigated the hypothesis that greater neighborhood physical disorder would be related to more safety concerns among participants. The second model examined whether greater neighborhood physical disorder would be related to poorer self-rated health. Finally, stratified regression coefficients were investigated to determine the presence of racial/ethnic differences in these hypothesized relationships. All models adjusted for age, sex, and highest educational degree as well as census tract concentrated disadvantage, population density, and racial/ethnic diversity.

## Results

### Participant description

The sample (*n* = 9,080) had an average age of 68 years and was 60% female. The majority of the sample were non-Hispanic Whites (*n* = 5,812), followed by non-Hispanic Blacks (*n* =1,905), and Hispanics (*n* =1,363). Overall, the HRS participants reported good/fair health and feeling somewhat safe in their neighborhoods. Interviewer ratings of neighborhood physical disorder was generally low. A description of the analytic variables reported separately by race/ethnicity can be found in Table 1.

**Table 1:** Descriptive statistics, full sample and stratified by race/ethnicity, [mean (SD)].

	**Full sample**	**Non-hispanic whites**	**Non-hispanic blacks**	**Hispanics**
	**(*n* = 9,080)**	**(*n* = 5,812)**	**(*n* = 1,905)**	**(*n* = 1,363)**
Perceived neighborhood safety	5.22 (1.82)	5.54 (0.02)	4.34 (0.06)	4.75 (0.07)
Physical neighborhood disorder	0.26 (0.75)	0.11 (0.01)	0.49 (0.02)	0.42 (0.03)
Self-rated health	3.08 (1.03)	3.28 (0.01)	2.89 (0.03)	2.83 (0.04)
Educational degree^a^				
GED	5.51%	5.01%	6.56%	8.06%
H.S. diploma	44.82%	47.85%	47.14%	32.80%
Two year degree	6.59%	6.55%	7.50%	11.83%
Four year degree	15.09%	18.35%	10.45%	18.81%
Master's degree	8.36%	11%	4.46%	11.02%
Professional degree	2.43%	3.33%	0.00%	0.00%
Concentrated disadvantage	−0.01 (0.84)	−0.36 (0.01)	0.69 (0.03)	0.42 (0.03)
Population density	5,270 (13,013)	2,27 (95.38)	7,377 (445)	10,700 (706)
Racial/ethnic diversity	0.38 (0.20)	0.34 (0.00)	0.44 (0.01)	0.40 (0.01)
Sex^b^	60%	65%	66%	61%
Age	67.55 (11.31)	70.01 (0.15)	64.97 (0.28)	64.10 (0.34)

*SD, standard deviation*.

a *Compared to no degree*.

b *Compared to Men*.

### Race/ethnicity, neighborhood disorder, perceived safety, and self-rated health

Results of the weighted linear regressions predicting perceived neighborhood safety are presented in Table 2. Among non-Hispanic Whites (coef. = −0.42, *p* < 0.001) and Hispanics (coef. = −0.24, *p* < 0.05), greater neighborhood physical disorder was significantly related to feeling less safe in one's neighborhood. The relationship between neighborhood physical disorder and perceived neighborhood safety was not significant among non-Hispanic Blacks. Among non-Hispanic Blacks and Hispanics, level of education was not statistically significantly related to perceived neighborhood safety. Results of the weighted linear regressions predicting self-rated health can be found in Table 3. Greater neighborhood physical disorder was significantly related to poorer self-rated health among non-Hispanic Whites (coef. = −0.15, *p* < 0.001), non-Hispanic Blacks (coef. = −0.13, *p* < 0.001), and Hispanics (coef. = −0.11, *p* < 0.05). Greater concentrated disadvantage was statistically significantly related to lower ratings of health, but only for non-Hispanic Whites. For non-Hispanic Whites and non-Hispanic Blacks, greater age was statistically significantly related to poorer health evaluations.

**Table 2:** Weighted linear regressions predicting perceived neighborhood safety by neighborhood physical disorder, [coefficient (SE)].

	**Non-hispanic whites** **(*n* = 5,812)**	**Non-hispanic blacks** **(*n* = 1,905)**	**Hispanics** **(*n* = 1,363)**
Physical neighborhood disorder	−0.42^***^ (0.07)	−0.13 (0.08)	−0.24^*^ (0.12)
Educational degree^a^			
GED	0.17 (0.17)	−0.39 (0.34)	0.37 (0.36)
High school diploma	0.36^**^ (0.12)	0.20 (0.18)	0.09 (0.22)
Two year degree	0.54^***^ (0.15)	0.23 (0.28)	0.36 (0.34)
Four year degree	0.54^***^ (0.13)	−0.01 (0.37)	0.42 (0.34)
Master's degree	0.55^***^ (0.14)	−0.03 (0.31)	1.11^***^ (0.33)
Professional degree	0.76^***^ (0.15)	−2.17^*^ (1.03)	0.69* (0.35)
Concentrated disadvantage	−0.61^***^ (0.05)	−0.34^***^ (0.08)	−0.51^***^ (0.13)
Population density	−0.00^***^ (0.00)	−0.00^***^ (0.00)	−0.00 (0.00)
Racial/ethnic diversity	−0.49^***^ (0.15)	−0.24 (0.40)	−1.43^***^ (0.43)
Age	−0.00 (0.00)	−0.00 (0.00)	0.00 (0.01)
Sex^b^	−0.20^***^ (0.05)	−0.31 (0.17)	−0.04 (0.18)

*SE, standard error*.

*Covariates: educational degree, concentrated disadvantage, population density, racial/ethnic diversity, age, & sex were adjusted in the model*.

a *Compared to no degree*.

b *Compared to Men*.

* *p < 0.05*.

** *p < 0.01*.

*** *p < 0.001*.

**Table 3:** Weighted linear regressions predicting self-rated health by neighborhood physical disorder, [coefficient (SE)].

	**Non-hispanic whites** **(*n* = 5,812)**	**Non-hispanic blacks** **(*n* = 1,905)**	**Hispanics** **(*n* = 1,363)**
Physical neighborhood disorder	−0.15^***^ (0.03)	−0.13^***^ (0.03)	−0.11^*^ (0.05)
Educational degree^a^			
GED	0.21^*^ (0.10)	−0.17 (0.14)	−0.17 (0.18)
High school diploma	0.56^***^ (0.06)	0.24^***^ (0.07)	0.29^***^ (0.09)
Two year degree	0.65^***^ (0.09)	0.43^***^ (0.12)	0.32^*^ (0.13)
Four year degree	0.83^***^ (0.07)	0.59^***^ (0.11)	0.15 (0.24)
Master's degree	0.90^***^ (0.08)	0.57^***^ (0.16)	0.64^**^ (0.22)
Professional degree	0.86^***^ (0.11)	1.28^***^ (0.19)	0.92^**^ (0.38)
Concentrated disadvantage	−0.16^***^ (0.03)	0.02 (0.03)	−0.11 (0.06)
Population density	−0.00 (0.00)	0.00 (0.00)	−0.00 (0.00)
Racial/ethnic diversity	0.05 (0.09)	0.01 (0.14)	0.35 (0.22)
Age	−0.01^***^ (0.00)	−0.01^***^ (0.00)	−0.00 (0.00)
Sex^b^	0.14^***^ (0.03)	−0.07 (0.06)	0.03 (0.08)

*SE, standard error*.

*Covariates: educational degree, concentrated disadvantage, population density, racial/ethnic diversity, age, & sex were adjusted in the model*.

a *Compared to no degree*.

b *Compared to Men*.

* *p < 0.05*.

** *p < 0.01*.

*** *p < 0.001*.

## Discussion

This study adds new knowledge in understanding racial/ethnic health disparities among older adults, particularly for the two largest health disparity populations in the US. Older adults may spend more time in their neighborhoods as they transition out of the workforce, thus rendering neighborhoods a potentially more important determinant of health at this point in the lifespan (33, 34). There are racial/ethnic differences in neighborhood quality and health, and the neighborhood disparities may explain the health disparities. Yet, few studies have investigated racial/ethnic differences in the link between neighborhood disorder and older adults' health. Results of these scant studies have sometimes found that neighborhood physical disorder is worse for psychosocial outcomes among non-Hispanic Whites than non-Hispanic Blacks. Some researchers argued that the racial/ethnic difference in the effect of neighborhood disorder is explained by non-Hispanic Whites finding disorder as more threatening to personal safety (22). However, that assumption has never been empirically tested. Thus, we set out to test this assumption by comparing racial/ethnic groups on the link between neighborhood physical disorder as rated by third parties on (1) perceived neighborhood safety and (2) a specific health outcome, self-rated health. One strength of the current analysis was use of a neighborhood physical disorder scale rated by third parties to minimize concerns regarding common source bias (13), as one of the primary outcomes investigated herein was neighborhood safety as rated by the participants. The present study found that, although greater neighborhood physical disorder was related to more safety concerns among non-Hispanic Whites and Hispanics, the same was not true for non-Hispanic Blacks. That said, greater neighborhood physical disorder was related to worse self-rated health among all racial/ethnic groups.

### Neighborhood physical disorder and safety

Neighborhood physical disorder can be measured or defined in different ways. For example, some researchers have used the term “physical decay” and have included items such as “cigarettes in the street,” “protest or political message grafitti”) (13, 35). The current study defined neighborhood physical disorder using the presence of vandalism, trash on the streets/yards, and abandoned cars that build upon another well-established definition (3). Despite these nuanced differences in the definition, the presence of neighborhood physical disorder is known to be associated with an array of health-related outcomes such as decreased physical fitness (14), and diminishes a sense of trust and security in the surrounding area (36). Less literature has examined the link between neighborhood physical disorder and health among diverse racial/ethnic groups.

The theories of Status discord (23) and Epidemiological paradox (24) both argue that non-Hispanic Whites are more threatened than non-Hispanic Blacks by signs of neighborhood physical disorder. To our knowledge, no formal investigation exists regarding whether or not racial/ethnic groups differ in their perceptions of safety in relation to neighborhood physical disorder to substantiate these claims. The present study is among the first to investigate this argument, and in support of this hypothesis, results indicated that non-Hispanic White and Hispanic residents embedded in areas with greater physical disorder reported significantly greater perceived neighborhood safety concerns. Although our findings somewhat support the Status Discord and Epidemiological Paradox theories, we were unable to further compare the two theories, as HRS does not contain measures of cognitive dissonance or resilience, the proposed mechanisms for the racial/ethnic differences posited in these theories.

### Neighborhood physical disorder and self-rated health

All three groups (non-Hispanic White, non-Hispanic Black, and Hispanic) reported significantly worse health if they were living in areas with more signs of neighborhood physical disorder. To our knowledge, this is among one of the first studies to investigate racial/ethnic differences in links between neighborhood physical disorder and health. The only other investigation similar to the present study was Echeverria's et al. (27) which examined neighborhood characteristics (e.g., neighborhood problems and neighborhood cohesion) and found no racial/ethnic differences in lifestyle behaviors (e.g., smoking, depression, drinking, and exercise activity). Our results are consistent with these past findings as there were no racial/ethnic differences in the disorder-self-rated health link. On average, non-Hispanic Whites live in relatively economically advantaged neighborhoods with less disorder than non-Whites (20, 37, 38). In addition, non-Hispanic Whites have better health compared to other racial/ethnic groups (39, 40). So, although we found that neighborhood physical disorder elicits more safety concerns among non-Hispanic Whites and Hispanics than non-Hispanic Blacks, our results clearly do not suggest that signs of disorder should only be ameliorated in primarily non-Hispanic White neighborhoods. Conversely, given no significant differences in the association between neighborhood physical disorder and self-reported health across the racial/ethnic groups assessed in the current study, our results suggest that community-level interventions targeting neighborhood physical disorder may not only improve community-level health, but minimize racial/ethnic health disparities. However, these neighborhood-level interventions should be dovetailed by policies that ensure equitable housing policies for affected communities. Established forms of interventions include modeling and strengthening a sense of community among neighbors in order to promote higher usage of public spaces and increasing attachment (41). Community organized efforts may also include the remediation of disordered environments (e.g., cleaning up litter, removing graffiti) (2, 14).

### Limitations and future directions

Health and Retirement Study is a national sample of US older adults. Although older adults have a lower likelihood of being victimized, they nevertheless report more safety concerns in their neighborhoods compared to younger adults (42). As such, findings from the present study may not generalize to younger samples of US residents. While the current study has many strengths, there are some notable limitations. First, HRS participants self-reported non-Hispanic White, non-Hispanic Black, and Hispanic status, and when these categories do not accurately capture a participant's racial/ethnic status, the participant selects “other.” The “other” category likely includes a diverse sample of individuals, including those who may be multi-racial, and were not included in the present study given the inability to make meaningful comparisons. Additionally, HRS lacks sufficient data to further disentangle different racial groups who each identify as Hispanic, and as such, those groups were equated for the purposes of the present analyses. Future studies with appropriate data may benefit from additional within- and between-group comparisons. Second, HRS lacks adequate data to operationalize other forms of disorder that have been identified in the neighborhood literature (e.g., social disorder, including drug use) and the pattern of relationships between neighborhood social disorder and various health outcomes, and racial/ethnic differences therein, may differ from that of neighborhood physical disorder. Third, both of the outcomes evaluated in the present study, perceived neighborhood safety and health, were self-reported. Although HRS collects data on classic single-item perceived neighborhood safety and self-rated health items, with the latter receiving substantial validation in published research (43), we acknowledge that these items are subjective, and more comprehensive measures of these constructs may increase reliability of the outcomes measured in the present study. Fourth, HRS does not ask participants how long they have lived in their current neighborhoods, precluding the ability to investigate whether participants may acclimate to signs of disorder in their neighborhoods, or whether the associations with disorder may accumulate over time in relation to residents' health. Lastly, future research should investigate objectively-assessed health outcomes to overcome potential self-report biases which may further vary systematically by race/ethnicity. Future research should incorporate more comprehensive scales of perceived neighborhood safety and health. Nevertheless, the current study is among the first to characterize the nuanced ways in which neighborhood physical disorder relates to people's subjective experiences of their environments and their health.

## Conclusion

The theoretical aim of the study was to examine the subjective experience of neighborhood physical disorder among a diverse group of older US adults, as neighborhood environments are viewed in the eyes of the beholder. The policy aim was to identify potential differential vulnerability to neighborhood physical disorder, which is a modifiable aspect of neighborhoods. The significant association between neighborhood physical disorder and self-rated health highlights that all racial/ethnic groups may benefit from neighborhood-level interventions. As racial/ethnic disparities continue to be a national concern, it is imperative to include interventions for those living in disordered areas. Feeling safe may promote health-promoting behaviors which can improve health and wellbeing for all members of society. Given that neighborhood factors such as neighborhood physical disorder is part and parcel of the social determinants of health framework, this work aligns with the Healthy People 2030 goal for promoting better health for all individuals, community health, and in turn improving the quality of life for all (Social determinants 2030) (44).

## Data availability statement

The original contributions presented in the study are included in the article/supplementary material, further inquiries can be directed to the corresponding author.

## Ethics statement

The studies involving human participants were reviewed and approved by University of Michigan, Institutional Review Board. The patients/participants provided their written informed consent to participate in this study.

## Author contributions

All authors listed have made a substantial, direct, and intellectual contribution to the work and approved it for publication.

## Funding

This research was based on work supported by a National Institutes of Health/National Institute on Aging Career Development Grant (4R00AG055699-03) to JR, Ph.D, and further supported by NIA (U01AG009740 and R21AG045625) to support Health & Retirement Study (HRS) data collection and the development of the HRS contextual data resource. Further, this research was also supported by Chapman University's Innovation in Diversity and Inclusion Research, Scholarship, and Creative Activity awarded to AV and JR. Not all data used in the present study are publicly available, but rather are accessible *via* a restricted data use agreement. Both public and restricted data are acquired *via* the HRS website (https://hrs.isr.umich.edu). These analyses were not preregistered.

## Conflict of interest

The authors declare that the research was conducted in the absence of any commercial or financial relationships that could be construed as a potential conflict of interest.

## Publisher's note

All claims expressed in this article are solely those of the authors and do not necessarily represent those of their affiliated organizations, or those of the publisher, the editors and the reviewers. Any product that may be evaluated in this article, or claim that may be made by its manufacturer, is not guaranteed or endorsed by the publisher.
